# Hidden burden of pneumococcal colonization in adults with viral respiratory infections: the role of influenza and RSV

**DOI:** 10.3389/fcimb.2026.1929747

**Published:** 2026-09-21

**Authors:** Rocío España-Bonilla, Sara Calvo-Silveria, Aida González-Díaz, Júlia Guevara-Figueras, Lucía Fernández-Delgado, Catalina Medina-Gómez, Laura Calatayud, Jordi Niubó, Mercè Gasa, Gabriela Abelenda-Alonso, M. Ángeles Domínguez, Judith Peñafiel, Carmen Ardanuy, Jordi Càmara

**Affiliations:** 1 Department of Clinical Microbiology, Bellvitge University Hospital -IDIBELL, L’Hospitalet de Llobregat, Spain; 2 Research Network for Respiratory Diseases (CIBERES-CB06/06/0037), ISCIII, Madrid, Spain; 3 Department of Pathology and Experimental Therapeutics, University of Barcelona, Barcelona, Spain; 4 Biostatistics Unit, Bellvitge Biomedical Research Institute-IDIBELL, L’Hospitalet de Llobregat, Spain; 5 Respiratory Department, Bellvitge Hospital Universitari de Bellvitge-IDIBELL, L’Hospitalet de Llobregat, Spain; 6 Department of Clinical Sciences, University of Barcelona, Barcelona, Spain; 7 Department of Infectious Diseases, Bellvitge University Hospital -IDIBELL, L’Hospitalet de Llobregat, Spain; 8 Research Network for Infectious Diseases (CIBERINFEC- CB21/13/00009), ISCIII, Madrid, Spain

**Keywords:** colonization, IAV infection, pneumococcal vaccines, *Streptococcus pneumoniae*, viral infection

## Abstract

**Introduction:**

Although pneumococcal colonization is uncommon in adults, it may increase during respiratory viral infections. We investigated the frequency of pneumococcal colonization, associated factors, and serotype distribution in adults with confirmed respiratory viral infection.

**Methods:**

We prospectively included adults presenting to the Emergency Department of Bellvitge University Hospital with influenza A virus (IAV), SARS-CoV-2, or respiratory syncytial virus (RSV) infection, as well as 33 virus-negative controls (December 2023–January 2024). Nasopharyngeal swabs were tested for *Streptococcus pneumoniae* using a RT-PCR assay (lytA and piaB genes). Clinical data were analysed using Cox proportional hazards regression models.

**Results:**

A total of 616 episodes were studied (IAV, n=391; SARS-CoV-2, n=162; RSV, n=28; >1 virus, n=35). Pneumococcal colonization was detected in 85 individuals, with the highest prevalence among IAV-infected patients (14.8%), followed by RSV (10.7%) and SARS-CoV-2 (9.3%). Individuals with viral infection showed higher pneumococcal loads (median lytA C_T_ 23.1 [18.7–27.4]) compared to those without infection (C_T_ 31.8 [31.5–33.3]). IAV (OR 3.52; 95%CI 1.30–9.17) and RSV (OR 4.04; 95%CI 1.11–13.50) were independently associated with pneumococcal colonization. Among adults <65 years, female sex (OR for male sex 0.43; 95% CI 0.19–0.90) was associated with colonization, whereas in those ≥65 years, RSV infection (OR 5.56; 95% CI 1.03–27.31) was the main association. Detected serotypes were diverse, predominantly non-PCV serotypes.

**Conclusions:**

Pneumococcal colonization was more frequent during IAV or RSV than SARS-CoV-2 infection. In older adults, RSV infection showed a particularly strong association, highlighting potential implications for pneumococcal and RSV vaccination strategies.

## 1 Introduction

*Streptococcus pneumoniae* infections are among the most frequent vaccine-preventable diseases worldwide (Toor et al., 2021). Pneumococcus colonizes the upper respiratory tract of humans, where it can persist and spread to other hosts. Colonization rates are high in children under 5 years old, reaching up to 50%, and are reported to be low in healthy adults (Weiser et al., 2018). Nevertheless, colonization rates in adults can be modified depending on some conditions such as chronic respiratory diseases, close contact with young children and the presence of respiratory viral infections (Almeida et al., 2021).

Interactions between pneumococci and viral pathogens can facilitate the colonization and the transmission of *S. pneumoniae*, making seasonal respiratory viral infections key drivers in the spread of pneumococcus (Manna et al., 2022). Viral infections, as well as other inflammatory conditions of the respiratory tract, facilitate pneumococcal colonization and increase bacterial load, resulting in synergistic interactions that can exacerbate disease severity (Zhou et al., 2012). Indeed, co-infection with *Streptococcus pneumoniae* and respiratory viruses is associated with complications of the lower respiratory tract and poorer clinical outcomes (Liu et al., 2021). Among these, the interaction between pneumococci and Influenza virus (IAV) is one of the most thoroughly documented examples of bacteria-virus interplay, with a well-established impact on disease progression (Wolter et al., 2014). IAV has been shown to promote *S. pneumoniae* colonization in murine models (Mina et al., 2014), primarily through alterations in lung physiology, such as epithelial damage and surfactant dysfunction, that may create a favourable environment for subsequent pneumococcus superinfection, contributing to increased respiratory complications (McCullers, 2014). Similarly, in a human challenge study, pneumococcal colonization was associated with impaired mucosal immune responses to IAV vaccination, potentially facilitating the development of more severe disease (Carniel et al., 2021).

Non-pharmacological COVID-19 containment measures, implemented to control the spread of SARS-CoV‑2, also led to a decrease in infections caused by other respiratory pathogens, including *S. pneumoniae* (Amin-Chowdhury et al., 2021). However, this reduction appears to reflect the decrease in seasonal viral infections rather than an actual reduction in pneumococcal carriage (Danino et al., 2022). In fact, the number of cases of pneumococcal invasive disease began to rise in several countries after relaxation of SARS-CoV‑2 control measures (Shaw et al., 2023; Rybak et al., 2024), returning to pre-pandemic levels in some regions (Hyams et al., 2023). In this context, the complex interplay between viral infections and pneumococcal diseases underscores the importance of ongoing surveillance and adaptive public health responses. For this reason, we aimed to explore the prevalence of pneumococcal colonization in adult patients presented to the emergency department with a respiratory viral infection due to IAV, SARS-CoV‑2 or respiratory syncytial virus (RSV). Specifically, our objectives were to analyse differences in the rate of pneumococcal colonization between viruses, describe the population of pneumococcal serotypes, and explore the clinical impact of pneumococcal colonization and vaccination status on viral respiratory infections.

## 2 Materials and methods

### 2.1 Study settings

This study was performed at Bellvitge University Hospital, a referral hospital that attends adult patients (≥18 years old) at the Metropolitan Area of Barcelona (Spain). All patients with suspected respiratory viral infection are routinely screened through the Alinity m Resp-4-Plex assay (Abbott Molecular, US) and positive samples are frozen for further studies. In this work, we analysed all nasopharyngeal swabs that tested positive for IAV, SARS-CoV-2 and/or RSV from patients admitted to the emergency department between December 18th (2023) and January 18th (2024). For the analysis of pneumococcal load through real-time PCR, a selection of 33 samples that tested negative for viral infection during the same period were studied. Only one episode per patient was considered.

### 2.2 Pneumococcal detection and serotyping

Nasopharyngeal samples were screened for the presence of *S. pneumoniae* as follows. DNA was extracted using the MagMAX™ Viral/Pathogen kit (KingFisher™ Flex purification system; Thermo Fisher Scientific). A multiplex real-time PCR (RT-PCR) was performed with the AgPath-ID one-step RT-PCR kit (Thermo Fisher Scientific) in a QuantStudio 5 thermocycler (ThermoFisher, UK) using *lytA* and *piaB* genes as targets, with primers and probes previously described (**Supplementary Table 1**) (Carvalho et al., 2007; Trzciński et al., 2013). A sample was considered positive for *S. pneumoniae* when both targets tested positive with a cycle threshold value (C_T_) lower than 38 cycles and differed by no more than 3 C_Ts_. Discordant results were resolved with a second RT-PCR targeting the *SP2020* gene. Samples without amplification or only one positive target were considered negative (Tavares et al., 2019). Pneumococcal serotyping was performed with the S. PneumoStrip (Operon^®^) commercial kit, which detects 76 serotypes based on their genetic sequences. The method involves reverse hybridization, where amplified serotype-specific DNA sequences bind to probes on nylon membranes and are detected with a streptavidin-peroxidase conjugate (Ercibengoa et al., 2021).

### 2.3 Clinical data

Clinical characteristics were retrospectively collected, including age, sex, toxic habits, comorbidities (diabetes, chronic liver disease, chronic heart disease, COPD, bronchiectasis, asthma, chronic renal failure, malignancies, immunosuppression and HIV infection), clinical presentation and 30-day mortality. Vaccination status regarding IAV, SARS-CoV-2 and *S. pneumoniae* was reviewed. For IAV, patients were considered vaccinated if received the seasonal 2023–2024 vaccine. Regarding pneumococcal vaccines, patients who received the PPSV23 polysaccharide vaccine within the last 5 years or a conjugate vaccine (PCV13 or PCV20) at any time but more than 14 days before the positive RT-PCR were considered vaccinated (Bonten et al., 2015). For SARS-CoV-2, all patients with at least one dose were considered vaccinated. Patients with no vaccination data in electronic records were considered unvaccinated.

### 2.4 Statistical analysis

To study the factors associated with pneumococcal colonization, Cox proportional hazard regression models were estimated in the group of individuals with viral infection. The models were adjusted by age, gender, smoking, COPD, comorbidities, viral infection and vaccination status. The analyses were repeated stratifying by age groups (<65 and ≥65 years old). To study the factors associated with mortality, cox regression for each factor was estimated. These models were repeated adjusted by age, sex, viral infection (IAV, SARS-CoV-2 and RSV), pneumococcal colonization, comorbidities (chronic heart disease, malignancies and immunosuppression), clinical presentation (mild disease, hospitalization and respiratory failure) and vaccination status (SARS-CoV-2, IAV and *S. pneumoniae* vaccination). Because hospitalization violated the proportional hazards assumption (Grambsch and Therneau, 1994), we applied a time-varying methodology to adress this issue (Therneau and Grambsch, 2000). *LytA* C_T_ values were compared between patients with and without viral coinfection using linear regression and visualized with violin plots. To assess the linear relation between *lytA* and *piaB* C_T_ values, Spearman correlation and CI95% via bootstrap method was calculated. For data processing and analysis, R Studio (v4.4.1) and Python (v.3.12) were used.

### 2.5 Ethics statements

This work has been approved by The Clinical Research Ethics Committee of Bellvitge University Hospital (PR280/22) and it is in accordance with the Declaration of Helsinki from the World Medical Association. Written informed consent was waived, as this was an observational study that used leftover isolates obtained during routine microbiological procedures. According to national normative data, all the data were anonymized, and patient confidentiality was always protected following the current legal normative in Spain (LOPD 3/2018 and RD 1720/2007).

## 3 Results

### 3.1 Patients with Influenza A virus infection have higher rates of pneumococcal colonization

Over the study period, 616 episodes of respiratory viral infection were analysed. The most frequent virus detected was IAV (n=391, 63.5%), followed by SARS-CoV‑2 (n=162, 26.3%) and RSV (n=28, 4.5%). No Influenza B virus was detected. Viral coinfection was detected in 35 episodes, mostly IAV plus SARS-CoV‑2 (n=30, 4.9%). Detection of SARS-CoV‑2 plus RSV (n=4, 0.6%) and IAV plus RSV (n=1, 0.2%) was infrequent. There were no statistically significant differences between the C_T_ values of the different viral infections, with median C_T_ values of 25.0 (IQR 19.0-29.0), 25.5 (IQR 18.0-32.2) and 26.0 (IQR 22.0-31.0) for IAV, SARS-CoV‑2 and RSV, respectively. The median age of the studied population was 72.0 years old (IQR 55.0-81.0), and 300 episodes (48.7%) occurred in females. Compared to SARS-CoV‑2, infections caused by IAV or RSV occurred in younger people, and more frequently required hospital admission, although no statistical differences were found (**Table 1**; **Supplementary Table 2****).** In contrast, SARS-CoV‑2 and RSV infections displayed higher rates of malignancies and immunosuppression. Higher 30-day mortality rates were observed in patients with SARS-CoV‑2 infection (6.8%).

**Table 1:** Clinical characteristics of the studied population by viral infection.

Clinical characteristics	Influenza A n=391	SARS-CoV-2 n=162	RSV n=28	>1 virus n=35	All n=616	*p*-value
Age, Median [Q1; Q3]	70.0 [51.0;80.0]	75.5 [61.5;83.0]	69.0 [56.5;74.0]	75.0 [57.0;85.0]	72.0 [55.0;81.0]	**0.002**
Female Sex	191 (48.8%)	79 (48.8%)	13 (46.4%)	17 (48.6%)	300 (48.7%)	0.996
Smoking	163 (41.7%)	66 (40.7%)	13 (46.4%)	12 (34.3%)	254 (41.2%)	0.787
Alcohol abuse	53 (13.6%)	11 (6.8%)	2 (7.1%)	3 (8.6%)	69 (11.2%)	0.120
Comorbidities						0.297
0	105 (26.9%)	34 (21.0%)	5 (17.9%)	3 (8.6%)	147 (23.9%)	
1	96 (24.6%)	43 (26.5%)	9 (32.1%)	12 (34.3%)	160 (26.0%)	
2	101 (25.8%)	52 (32.1%)	6 (21.4%)	11 (31.4%)	170 (27.6%)	
>2	89 (22.8%)	33 (20.4%)	8 (28.6%)	9 (25.7%)	139 (22.6%)	
Diabetes	98 (25.1%)	38 (23.5%)	6 (21.4%)	10 (28.6%)	152 (24.7%)	0.895
Chronic liver disease	17 (4.3%)	12 (7.4%)	1 (3.6%)	4 (11.4%)	34 (5.5%)	0.160
Chronic heart disease	136 (34.8%)	35 (21.6%)	9 (32.1%)	13 (37.1%)	193 (31.3%)	**0.020**
COPD	74 (18.9%)	24 (14.8%)	2 (7.1%)	9 (25.7%)	109 (17.7%)	0.171
Bronchiectasis	8 (2.0%)	3 (1.9%)	1 (3.6%)	0 (0.0%)	12 (1.9%)	0.723
Asthma	53 (13.6%)	12 (7.4%)	4 (14.3%)	7 (20.0%)	76 (12.3%)	0.077
Chronic renal failure	75 (19.2%)	23 (14.2%)	3 (10.7%)	11 (31.4%)	112 (18.2%)	0.067
Malignancies	54 (13.8%)	43 (26.5%)	8 (28.6%)	6 (17.1%)	111 (18.0%)	**0.002**
Immunosuppression	82 (21.0%)	70 (43.2%)	15 (53.6%)	8 (22.9%)	175 (28.4%)	**<0.001**
HIV	6 (1.5%)	1 (0.6%)	0 (0.0%)	1 (2.9%)	8 (1.3%)	0.557
Clinical presentation						
Mild disease	31 (7.9%)	23 (14.2%)	1 (3.6%)	0 (0.0%)	55 (8.9%)	**0.015**
Pneumonia	76 (19.4%)	34 (21.0%)	6 (21.4%)	10 (28.6%)	126 (20.5%)	0.635
Hospitalization	157 (40.2%)	50 (30.9%)	13 (46.4%)	13 (37.1%)	233 (37.8%)	0.163
ICU admission	8 (2.0%)	0 (0.0%)	1 (3.6%)	0 (0.0%)	9 (1.5%)	0.146
Respiratory failure	71 (18.2%)	36 (22.2%)	9 (32.1%)	6 (17.1%)	122 (19.8%)	0.251
Pleural effusion	6 (1.5%)	3 (1.9%)	1 (3.6%)	1 (2.9%)	11 (1.8%)	0.423
Sepsis	5 (1.3%)	2 (1.2%)	1 (3.6%)	2 (5.7%)	10 (1.6%)	0.135
Vaccination status						
SARS-CoV-2	351 (89.8%)	147 (90.7%)	27 (96.4%)	32 (91.4%)	557 (90.4%)	0.987
Seasonal Influenza	151 (38.6%)	75 (46.3%)	10 (35.7%)	9 (25.7%)	245 (39.8%)	0.265
PCV	59 (15.1%)	40 (24.7%)	3 (10.7%)	6 (17.1%)	108 (17.5%)	**0.048**
Outcome						
30-day mortality	15 (3.8%)	11 (6.8%)	1 (3.6%)	4 (11.4%)	31 (5.0%)	0.124

Data is shown as number of cases and percentage, otherwise stated. Statistically significantvalues are highlighted in bold. Complete data by age group is included in **Supplementary Table 2**.Statistically significant values are highlighted in bold.

Regarding pneumococcal colonization, it was detected in 85 samples (13.8%). Of these, 83 tested positive for *lytA* and *piaB* genes and 2 for *lytA* and *SP2020*. The mean C_T_ value for *lytA* was 23.1 (SD 5.1) and for *piaB* 23.1 (SD 5.2), with very strong correlation (ρ=0.97; 95%CI 0.94-0.98) (**Supplementary Figure 1**). The rate of *S. pneumoniae* detection by viral infection is shown in **Figure 1**. Pneumococcal colonization was higher in patients infected with IAV (14.8%) compared to those with SARS-CoV‑2 (9.3%; OR 1.71, ρ=0.097) and RSV (10.7%; OR 1.45, ρ=0.604), although these differences were not statistically significant. Similarly, no significant difference was observed when comparing SARS-CoV-2 to RSV (OR 0.85, ρ=1.0). The rate of pneumococcal colonization increased to 25.7% in episodes of viral co-infection. Regarding IAV and SARS-CoV‑2 co-infection, the rate of colonization was 20% (6/30). By age group, young adults (18–64 years; 15.3%) exhibited higher pneumococcal colonization rates compared with older adults (≥65 years; 12.1%, ρ=0.15). Nevertheless, older adults showed lower median *lytA* C_T_ values (21.65 [IQR 17.81-27.22] vs 24.13 [IQR 20.95-28.09]).

**Figure 1:**
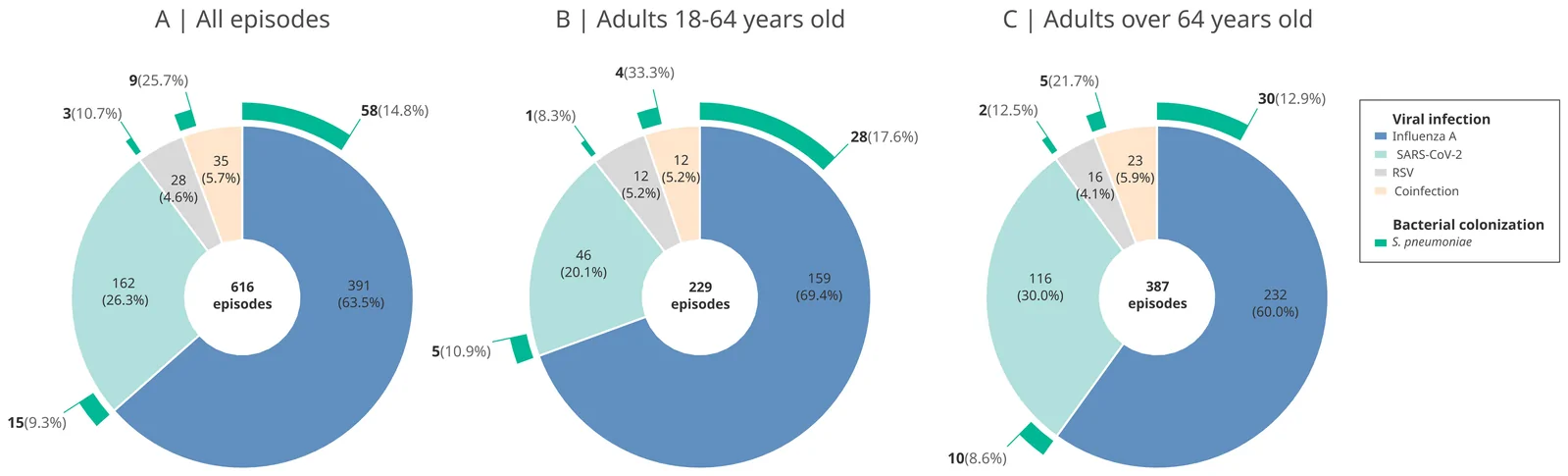
Pneumococcal colonization by viral infection and age group. **(A)** All episodes. **(B)** Adults 18-64 years old. **(C)** Adults over 64 years old. Coinfection refers to the detection of two or more viruses in a single sample.

We also analyzed the *lytA* C_T_ values in samples with pneumococcal detection according to the viral infection (**Figure 2**). Overall, samples in which a virus was detected had significantly lower median *lytA* C_T_ values than those from the control group with no viral detection (23.1 [IQR 18.7-27.4] vs 31.8 [IQR 31.5-33.3], *p* < 0.001), indicating a higher pneumococcal load. Regarding viral infections, patients with RSV (19.0 [IQR 18.7-20.6]) and Influenza A (22.8 [IQR 18.4-27.0]) showed higher pneumococcal colonization load than those with SARS-CoV-2 (26.4 [IQR 20.5-27.9]). However, no statistically significant differences in *lytA* C_T_ values across the different viral infections were observed.

**Figure 2:**
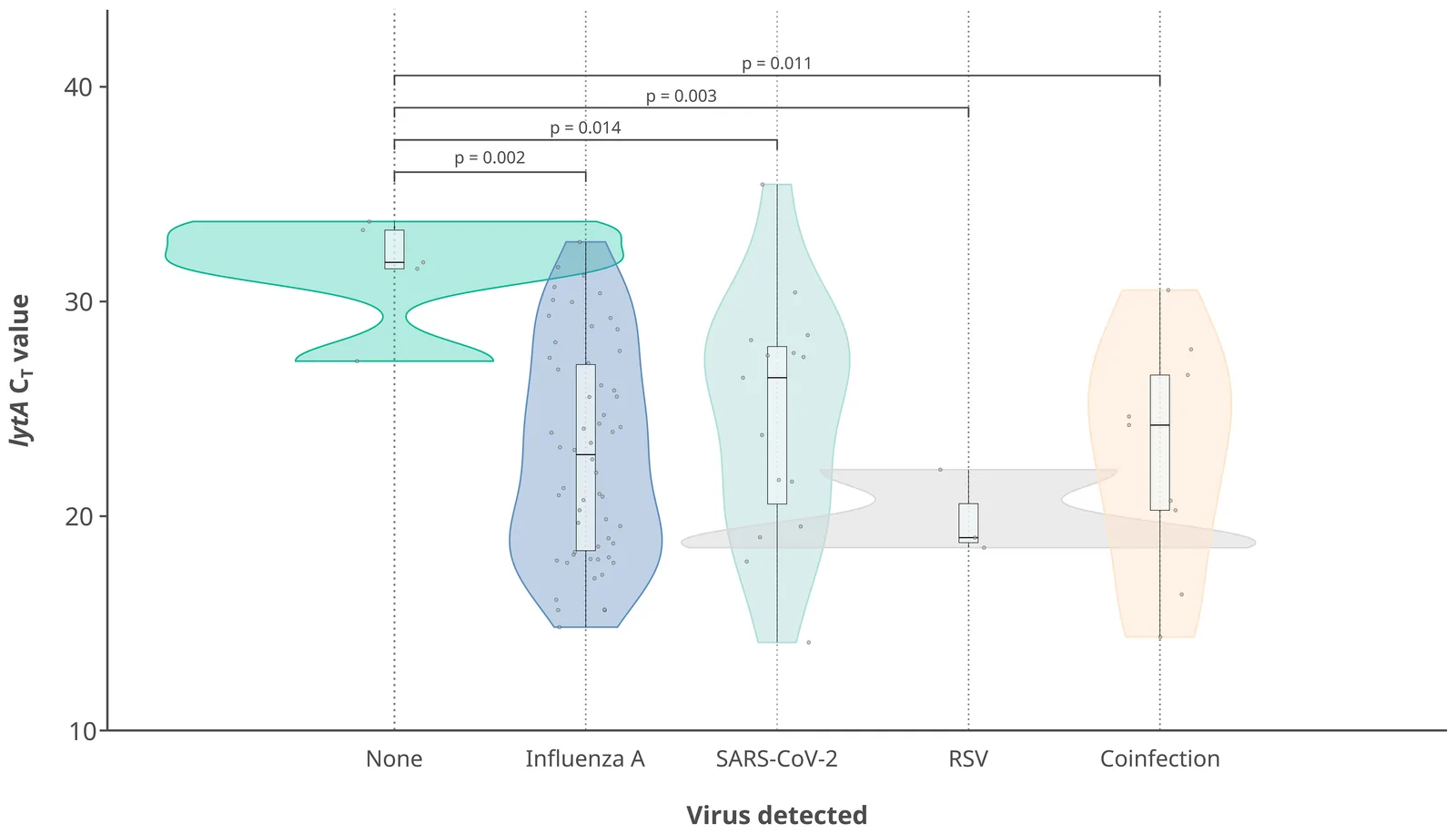
Pneumococcal load by viral infection. The figure shows the distribution of *lytA* C_T_ values, and a density plot stratified by type of viral infection. Episodes in which no virus was detected were classified in the “None” group (control group). Comparisons of mean *lytA* C_T_ values between groups were performed. The figure shows the corresponding *p-*values comparing the “None” group with each viral infection category. No statistically significant differences were observed in *lytA* C_T_ values between the different viral infections.

### 3.2 Influenza A and RSV are associated with pneumococcal colonization, but their importance differs across age groups

To identify factors associated with pneumococcal colonization, we estimated different Cox proportional hazard regression models (**Figure 3**; **Supplementary Table 3**). In the overall population, IAV and RSV were statistically associated with pneumococcal colonization, and this was consistent in all tested models. Odds ratios for IAV and RSV infections were 3.52 (95%CI 1.30-9.17) and 4.04 (95%CI 1.11-13.50), respectively. No other factors showed statistically significant results. Interestingly, when factors were analysed by age group, the results differed notably. Among young adults, female sex (OR 0.43 for male sex [95%CI 0.19-0.90]) was clearly associated with increased rates of pneumococcal colonization. Among viral infections, IAV had the highest odds of pneumococcal colonization, although this association was not statistically significant (OR 3.70 [95%CI 0.69-18.90]). In contrast, among older adults, RSV infection was the only factor significantly associated with pneumococcal colonization (OR 5.56 [95%CI 1.03-27.31]). In this group of patients, IAV infection showed a tendency toward increased colonization (OR 2.94 [95%CI 0.76-10.38]), whereas female sex appeared to have minimal influence (OR for male sex 0.95 [95%CI 0.45-1.96]). Notably, pneumococcal vaccination with PCV was not associated with reduced rates of pneumococcal colonization across any age group.

**Figure 3:**
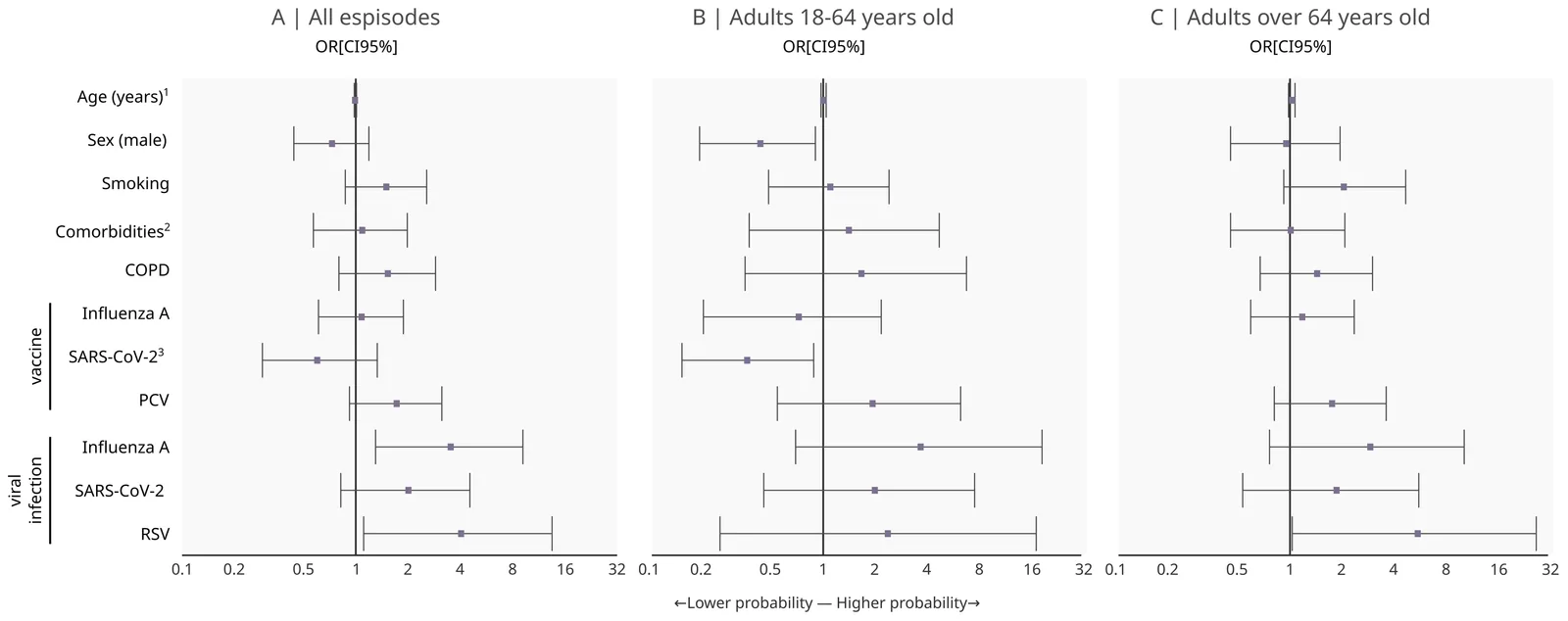
Factors associated with pneumococcal colonization by age group. **(A)** All episodes. **(B)** Adults 18-64 years old. **(C)** Adults over 64 years old. Estimations were performed through logistic regression for each factor. ^1^Age was included in the model as a continuous variable. ^2^Comparisons were performed between patients with 0–2 comorbidities and those with more than 2 comorbidities, excluding COPD. ^3^SARS-CoV-2 vaccination could not be included in the group of individuals ≥65 years old due to insufficient values of unvaccinated people. Additional models are included in **Supplementary Material**, **Table S3**.

We also analyzed the association between pneumococcal colonization and 30-day mortality (**Supplementary Table 4**; **Supplementary Figure 2**). The presence of chronic heart disease (HR 3.06 [95%CI 1.29-7.27]), immunosuppression (HR 2.77 [95%CI 1.22-6.29] and respiratory failure (HR 4.83 [95%CI 1.93-12.1]) were all significantly associated with 30-day mortality. In contrast, pneumococcal colonization was not associated with mortality in our study population (HR 0.68 [95%CI 0.22-2.05]). Among viral infections, only SARS-CoV-2 was statistically associated with increased 30-day mortality (HR 6.08 [95%CI 1.86-19.8]), although IAV also showed a trend toward higher mortality (HR 3.63 [95%CI 1.00-13.2]).

### 3.3 Pneumococcal serotypes were diverse, with predominance of serotypes not covered by current PCVs and associated with low invasiveness

Of the 85 samples that tested positive for *S. pneumoniae*, serotype could be determined in 75. More than one serotype was detected in 18 samples. The distribution of pneumococcal serotypes detected is shown in **Figure 4**. The most common serotype was serotype 23A (n=11 patients), followed by serotype 3 (n=9 patients). A significant proportion of patients (n=27, 36.0%) carried at least one serotype not covered by any currently available PCVs. Among these, full serotype coverage would potentially be achieved by PCV21-V116 in 43 patients (57.3%) and by PCV20 in 24 patients (32.0%).

**Figure 4:**
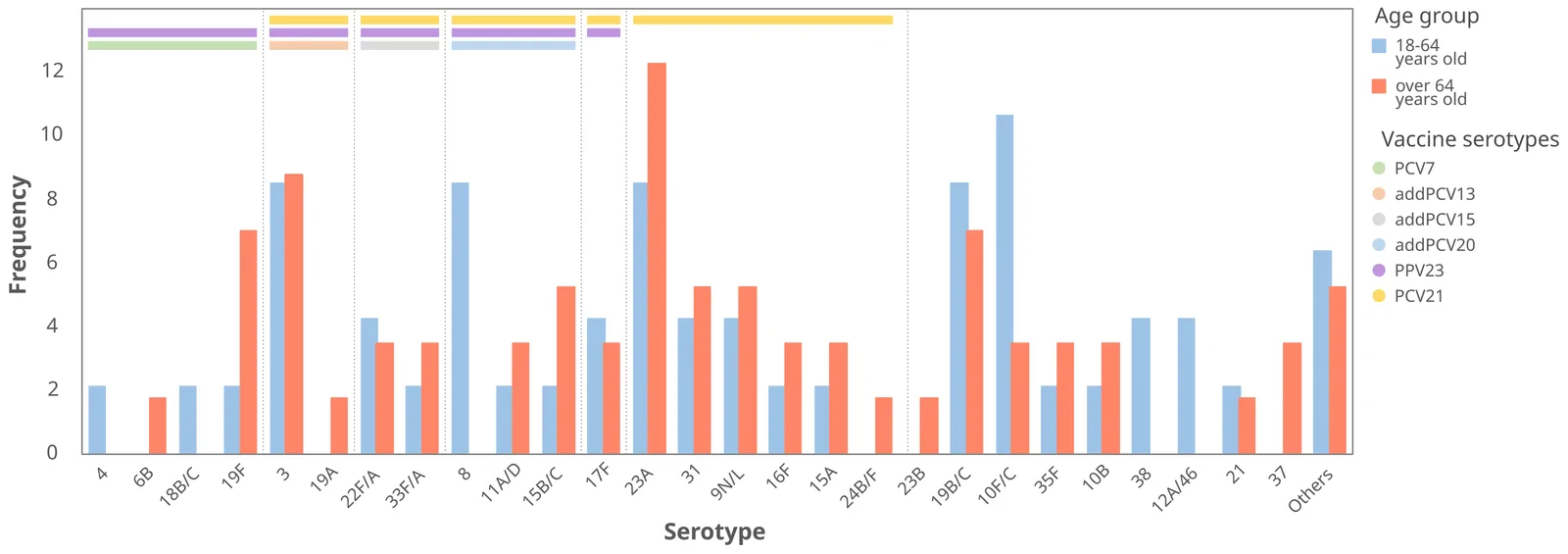
Distribution of pneumococcal serotypes by age group. The figure displays the frequency of detected serotypes, regardless of whether some were identified in only a single patient. The group “others” include serotypes not included in PCVs and that were detected only once. Upper horizontal lines indicate the different pneumococcal vaccines in which serotypes are included: PCV7; addPCV13: serotypes added by PCV13; addPCV15: serotypes added by PCV15; addPCV20: serotypes added by PCV20; PPSV23: 23-valent pneumococcal polysaccharide vaccine. PCV21: 21-valent PCV.

When analyzing the predominant serotypes responsible for invasive pneumococcal disease (IPD) in adults in Spain (3, 8, and 22F) (Pérez-García et al., 2024), it is noteworthy that serotype 8 was detected exclusively in young adults, whereas serotypes 3 and 22F were observed at similar frequencies in both young and older adults. Quantitative assessment of pneumococcal load, based on median *lytA* C_T_ values, revealed that serotype 3 was associated with the highest bacterial load (24.3 [IQR 21.0-27.7), followed by serotype 8 (26.04 [IQR 24.3-27.8]) and serotype 22F (26.9 [IQR 23.1-31.3]). Among older adults who were appropriately vaccinated with either PCV13 or PCV20 (n = 77, 19.9%), 13 were colonized by *S. pneumoniae* (13/77, 16.9%) and 5 carried serotypes included in the administered PCV formulation. Serotypes detected were 3 (n=3; 1 individual vaccinated with PCV13 and 2 with PCV20), 19A (n=1, 1 individual vaccinated with PCV20) and 19F (n=1, 1 individual vaccinated with PCV20).

## 4 Discussion

Inflammatory conditions of the upper respiratory tract, such as viral infections, can promote the colonization of *S. pneumoniae*, which in turn may exacerbate the severity of the viral infection. This could partly explain the increased detection of *S. pneumoniae* during winter seasons, corresponding with higher circulation of respiratory viruses. The interaction between pneumococcus and viral infections has been classically described in the context of IAV infection, with evidence from murine models demonstrating increased pneumococcal load, supported by clinical data showing enhanced disease severity (Diavatopoulos et al., 2010; Mina et al., 2014; Wolter et al., 2014). Nevertheless, evidence of interactions between SARS-CoV-2 and *S. pneumoniae* remains limited. In fact, following the emergence of the SARS-CoV-2 pandemic, a global decrease in the incidence of pneumococcal diseases was observed, also in our area (Amin-Chowdhury et al., 2021; Calvo-Silveria et al., 2025). In this study, we explored the frequency of pneumococcal colonization and the factors influencing it in adult patients with respiratory infection caused by IAV, SARS-CoV-2, or RSV.

Sample collection was conducted in Catalonia during the 2023–2024 winter season, coinciding with a high circulation of IAV. This was, in fact, the first winter season with substantial circulation of respiratory viruses other than SARS-CoV-2 since the onset of the COVID-19 pandemic. During the sample collection period, the estimated incidence of IAV infections ranged from 213 to 358 episodes per 100,000 population (https://sivic.salut.gencat.cat/, accessed July 2^nd^ 2026), which was significantly higher than the estimated incidence of SARS-CoV-2 infections (70 to 156 episodes per 100,000 population). Therefore, it is coherent that IAV was the most prevalent virus detected. Regarding clinical data, differences based on the type of viral infection were observed, with patients infected with SARS-CoV-2 being older and exhibiting a higher prevalence of malignancies and immunosuppression. The observation that IAV infections affected younger individuals with fewer severe comorbidities might point to a greater severity of illness associated with IAV compared to SARS-CoV-2, as previously reported (Xue et al., 2022). However, it should be noted that the vaccination rate for SARS-CoV-2 was significantly higher than that for seasonal Influenza, which likely contributed to a greater proportion of mild infections. Indeed, more than 80% of vaccinated individuals (451/557) had received three or more doses of the SARS-CoV-2 vaccine. This discrepancy not only highlights the success of SARS-CoV-2 vaccination programs in mitigating the impact of the COVID-19 pandemic but also underscores the urgent need to enhance seasonal Influenza vaccination uptake.

Pneumococcal colonization is the critical step in the pathogenesis of pneumococcal diseases. While colonization rates are high among children under 5 years, they are substantially lower in adults (Palmu et al., 2012; Narciso et al., 2025). Despite lower colonization rates, older adults experience a high incidence of pneumococcal disease, which is partly attributable to age-related immunosuppression and the higher prevalence of chronic medical conditions (Grant et al., 2023). Nevertheless, our findings show not only an increased frequency of pneumococcal colonization among adults, which may reflect a population bias resulting from the overrepresentation of individuals with comorbidities, but also an elevated pneumococcal load in the context of viral infection. These findings could provide insight into the pathogenesis of pneumococcal pneumonia in adults, given that respiratory viral infections enhance pneumococcal overgrowth, predisposing the host to bacterial lung invasion and pneumonia development (Zhou et al., 2012). Moreover, this represents a potential source of pneumococcal transmission to other individuals in the community highlighting the role of adults in the maintenance and transmission of pneumococci. Interestingly, our study identified a high pneumococcal colonization rate among young adults, a population generally characterized by a low incidence of pneumococcal disease. In our cohort, a substantial proportion of young patients were immunosuppressed or presented with comorbid conditions. Additionally, smoking and alcohol abuse, recognized risk factors for pneumococcal disease among young adults (Grau et al., 2014), were notably prevalent within this population. These findings indicate that patients requiring healthcare due to acute respiratory viral infections, including older adults and young adults with comorbidities or risk habits, exhibit higher rates of pneumococcal colonization than the general population. Consequently, these groups represent important targets for respiratory disease prevention, particularly through vaccination.

Regarding the viral-bacteria interactions, our data show a higher proportion of adults with pneumococcal colonization among infections caused by IAV or RSV. The pneumococcal load associated with IAV and RSV was also higher than that observed for SARS-CoV-2 infections. This suggests that interactions between pneumococci and respiratory viruses may be less significant in the case of SARS-CoV-2. Additionally, we observed notable age-related differences in the factors associated with pneumococcal colonization. Among young adults, female sex and infection with IAV virus were the factors most strongly associated with pneumococcal colonization. The higher propensity for pneumococcal colonization observed among females may reflect increased contact with children, a well-established risk factor for pneumococcal carriage (Wyllie et al., 2024; Almeida et al., 2025). In older adults, RSV infection was notably associated with an increased likelihood of pneumococcal colonization. Indeed, both IAV and RSV infections have previously been linked to the resurgence of pneumococcal diseases following the relaxation of non-pharmaceutical interventions due to the COVID-19 pandemic (Rybak et al., 2024). On the contrary, reported data seem to indicate that the rates of pneumococcal colonization remained stable (Danino et al., 2022; Wyllie et al., 2023). All these data suggest that the incidence of pneumococcal disease depends more on the triggering effect of IAV and RSV in promoting pneumococcal infection, rather than on a significant impact on colonization rates. The fact that pneumococcal and RSV interaction was observed mainly among older adults suggests that RSV may require additional host factors, such as more severe comorbidities, compared to IAV, to cause disease in the adult population (Ackerson et al., 2019). In any case, infections caused by IAV, RSV, or SARS-CoV-2 were associated with higher pneumococcal loads compared to cases without these viruses, suggesting that all three may contribute to promoting pneumococcal colonization (Brito-Mutunayagam et al., 2025). These findings underscore the importance of coordinated vaccination campaigns targeting both respiratory virus and *S. pneumoniae* to help mitigate the burden of epidemic peaks of respiratory infections in adults.

Over the past years, multiple pneumococcal conjugate vaccines (PCVs) have been introduced, mainly focused on paediatric immunization. Since 2023, several Autonomous Communities in Spain, including Catalonia, have recommended the use of the PCV20 for adults aged 65 years and older, as well as for individuals at increased risk. Our data show a low uptake of PCVs (<20%) among individuals aged 65 years and older, likely because the sample was collected during the first year of the program’s implementation. Regarding PCVs and viral infections, published evidence suggests that PCVs may have a beneficial role in preventing respiratory tract infections of viral origin, particularly those caused by IAV and SARS-CoV-2 (Lewnard et al., 2022; Lewnard et al., 2023). In our cohort, immunization with PCVs did not appear to influence overall pneumococcal colonization. Moreover, the identified serotypes were heterogeneous, with a predominance of non-PCV types and a high proportion of serotypes associated with carriage rather than invasiveness, such as serotypes 23A and 19F (Sá-Leao et al., 2011). These findings are consistent with our data, which show no impact of pneumococcal colonization on mortality. The beneficial effect of PCVs may therefore be attributable to a reduction in pneumococcal serotypes with higher invasive potential, most included in current PCV formulations, rather than to an effect on overall colonization. Nevertheless, since it has been reported that new PCVs covering additional serotypes may provide less protection against colonization, the re-emergence of invasive serotypes should be continuously monitored (Wong et al., 2025). In this regard, it is important to note that serotype 3 was among the most frequently detected serotypes, and its prevalence was markedly higher than what is typically reported in children (Siegel et al., 2014). In fact, it is a serotype typically found in adults, and their role in its transmission has been suggested (Safadi et al., 2025). This, together with its persistence as one of the most frequent causes of severe pneumococcal disease (Calvo-Silveria et al., 2024) underscores the need for the development of new adult pneumococcal vaccines with enhanced effectiveness against this serotype.

Our study contributes to the understanding of interactions between pneumococci and viral infections; however, it has several limitations that should be acknowledged. First, the methodology used for serotyping could not resolve all pneumococcal positive samples and in some cases the result could only indicate serogroup. Additionally, because analyses were performed directly on clinical samples without access to the corresponding pneumococcal strains, the presence of other encapsulated *Streptococcus mitis* group isolates could have affected the serotyping results. These limitations were addressed in the case of pneumococcal detection through the use of duplex PCR and the application of a C_T_ difference range. Second, since data were collected from a single geographical area, the generalizability of the results to other clinical settings should be evaluated. Nevertheless, this also allowed us to standardize the data collection and interpretation. Third, the retrospective nature of the clinical data collection did not allow us to analyse other factors that could favour pneumococcal colonization, such as contact with children or occupational exposure. Fourth, this is a cross-sectional study, and longitudinal studies analysing the dynamics of pneumococcal colonization during viral infections could provide valuable insights into the pathogenesis of pneumococcal diseases. Finally, patient recruitment was restricted to a one-month period that coincided with the peak circulation of IAV and RSV in our region. As only adults were included, RSV infections may have been underrepresented despite high community circulation. Furthermore, the study design did not capture the full seasonal dynamics of respiratory viruses, particularly SARS-CoV-2, whose circulation extends throughout the year. Therefore, the observed viral distribution may not be fully representative of the entire respiratory virus season.

In conclusion, our findings indicate that a significant proportion of adults with viral respiratory infections are colonized by *S. pneumoniae.* Our data also indicate that pneumococcal colonization was more associated with IAV or RSV than with SARS-CoV-2, although individuals with any viral infection showed higher pneumococcal loads than those without. Factors driving pneumococcal colonization vary by age, with female sex being a key determinant in young adults and RSV infection playing an important role in older adults. These results highlight the importance of integrated vaccination strategies targeting both respiratory viruses and *S. pneumoniae* to mitigate the impact of epidemic peaks of respiratory infections in the adult population.

## Data Availability

The raw data supporting the conclusions of this article will be made available by the authors, without undue reservation.

## References

[B1] Ackerson B. Tseng H. F. Sy L. S. Solano Z. Slezak J. Luo Y. . (2019). Severe morbidity and mortality associated with respiratory syncytial virus versus influenza infection in hospitalized older adults. Clin. Infect. Dis. 69, 197–203. doi: 10.1093/cid/ciy991 30452608 PMC6603263

[B2] Almeida S. T. Paulo A. C. Froes F. De Lencastre H. Sá-Leão R. (2021). Dynamics of pneumococcal carriage in adults: A new look at an old paradigm. J. Infect. Dis. 223, 1590–1600. doi: 10.1093/infdis/jiaa558 32877517

[B3] Almeida S. T. Paulo A. C. Simões A. S. Ferreira B. Sá-Leão R. (2025). *Streptococcus pneumoniae* carriage in adults during the COVID-19 pandemic in Portugal: Dominance of serotypes included in broader PCVs and of serotype 3. mSphere 10 (7), e00082-25. doi: 10.1128/msphere.00082-25 40492709 PMC12306169

[B4] Amin-Chowdhury Z. Aiano F. Mensah A. Sheppard C. L. Litt D. Fry N. K. . (2021). Impact of the Coronavirus Disease 2019 (COVID-19) pandemic on invasive pneumococcal disease and risk of pneumococcal coinfection with severe acute respiratory syndrome coronavirus 2 (SARS-CoV-2): Prospective national cohort study, England. Clin. Infect. Dis. 72, E65–E75. doi: 10.1093/cid/ciaa1728 33196783 PMC7717180

[B5] Bonten M. J. M. Huijts S. M. Bolkenbaas M. Webber C. Patterson S. Gault S. . (2015). Polysaccharide conjugate vaccine against pneumococcal pneumonia in adults. N. Engl. J. Med. 372, 1114–1125. doi: 10.1056/nejmoa1408544 25785969

[B6] Brito-Mutunayagam S. Hamilton D. O. Mitsi E. Solórzano C. Li G. De Macedo B. R. . (2025). Understanding the interaction of upper respiratory tract infection with respiratory syncytial virus and *Streptococcus pneumoniae* using a human challenge model: A multicenter, randomized controlled study protocol. PloS One 20, e0325149. doi: 10.1371/JOURNAL.PONE.0325149 40591700 PMC12212582

[B7] Calvo-Silveria S. González-Díaz A. Grau I. Marimón J. M. Cercenado E. Quesada M. D. . (2024). Evolution of invasive pneumococcal disease by serotype 3 in adults: A Spanish three-decade retrospective study. Lancet Regional Health - Europe 41, 100913. doi: 10.1016/j.lanepe.2024.100913 38737571 PMC11087702

[B8] Calvo-Silveria S. González-Díaz A. Marimón J. M. Cercenado E. Quesada M. D. Casabella A. . (2025). Resilience and emergence of pneumococcal serotypes and lineages in adults post-PCV13 in Spain: A multicentre study. J. Infect. Public Health 18 (1), 102619. doi: 10.1016/j.jiph.2024.102619 39662160

[B9] Carniel B. F. Marcon F. Rylance J. German E. L. Zaidi S. Reiné J. . (2021). Pneumococcal colonization impairs mucosal immune responses to live attenuated influenza vaccine. JCI Insight 6 (4), e141088. doi: 10.1172/jci.insight.141088 33497364 PMC7934923

[B10] Carvalho M. D. G. S. Tondella M. L. McCaustland K. Weidlich L. McGee L. Mayer L. W. . (2007). Evaluation and improvement of real-time PCR assays targeting lytA, ply, and psaA genes for detection of pneumococcal DNA. J. Clin. Microbiol. 45, 2460–2466. doi: 10.1128/JCM.02498-06 17537936 PMC1951257

[B11] Danino D. Ben-Shimol S. Van Der Beek B. A. Givon-Lavi N. Avni Y. S. Greenberg D. . (2022). Decline in pneumococcal disease in young children during the Coronavirus Disease 2019 (COVID-19) pandemic in Israel associated with suppression of seasonal respiratory viruses, despite persistent pneumococcal carriage: A prospective cohort study. Clin. Infect. Dis. 75, E1154–E1164. doi: 10.1093/cid/ciab1014 34904635 PMC8754767

[B12] Diavatopoulos D. A. Short K. R. Price J. T. Wilksch J. J. Brown L. E. Briles D. E. . (2010). Influenza A virus facilitates *Streptococcus pneumoniae* transmission and disease. FASEB J. 24, 1789–1798. doi: 10.1096/fj.09-146779 20097876

[B13] Ercibengoa M. Perez-Gavilan A. Alonso M. Azcue N. Cilla G. Marimón J. M. (2021). Comparison of four methods for *Streptococcus pneumoniae* capsular typing. Clin. Lab. 67, 1598–1602. doi: 10.7754/Clin.Lab.2020.201029 34258972

[B14] Grambsch P. M. Therneau T. M. (1994). Proportional hazards tests and diagnostics based on weighted residuals. Biometrika 81, 515–526. doi: 10.1093/biomet/81.3.515

[B15] Grant L. R. Meche A. McGrath L. Miles A. Alfred T. Yan Q. . (2023). Risk of pneumococcal disease in US adults by age and risk profile. Open Forum Infect. Dis. 10 (5), ofad192. doi: 10.1093/ofid/ofad192 37180598 PMC10167987

[B16] Grau I. Ardanuy C. Calatayud L. Schulze M. H. Liñares J. Pallares R. (2014). Smoking and alcohol abuse are the most preventable risk factors for invasive pneumonia and other pneumococcal infections. Int. J. Infect. Dis. 25, 59–64. doi: 10.1016/j.ijid.2013.12.013 24853638

[B17] Hyams C. Challen R. Hettle D. Amin-Chowdhury Z. Grimes C. Ruffino G. . (2023). Serotype distribution and disease severity in adults hospitalized with *Streptococcus pneumoniae* infection, Bristol and Bath, UK, 2006–2022. Emerg. Infect. Dis. 29, 1953–1964. doi: 10.3201/eid2910.230519 37735739 PMC10521591

[B18] Lewnard J. A. Bruxvoort K. J. Fischer H. Hong V. X. Grant L. R. Jódar L. . (2022). Prevention of Coronavirus Disease 2019 among older adults receiving pneumococcal conjugate vaccine suggests interactions between *Streptococcus pneumoniae* and severe acute respiratory syndrome coronavirus 2 in the respiratory tract. J. Infect. Dis. 225, 1710–1720. doi: 10.1093/infdis/jiab128 33693636 PMC7989304

[B19] Lewnard J. A. Bruxvoort K. J. Hong V. X. Grant L. R. Jódar L. Cané A. . (2023). Effectiveness of pneumococcal conjugate vaccination against virus-associated lower respiratory tract infection among adults: A case-control study. J. Infect. Dis. 227, 498–511. doi: 10.1093/infdis/jiac098 35323906 PMC9383607

[B20] Liu Y. Ling L. Wong S. H. Wang M. H. Fitzgerald J. R. Zou X. . (2021). Outcomes of respiratory viral-bacterial co-infection in adult hospitalized patients. EClinicalMedicine 37, 100955. doi: 10.1016/j.eclinm.2021.100955 34386745 PMC8343259

[B21] Manna S. McAuley J. Jacobson J. Nguyen C. D. Ullah M. Sebina I. . (2022). Synergism and antagonism of bacterial-viral coinfection in the upper respiratory tract. mSphere 7 (1), e00984-21. doi: 10.1128/msphere.00984-21 35044807 PMC8769199

[B22] McCullers J. A. (2014). The co-pathogenesis of influenza viruses with bacteria in the lung. Nat. Rev. Microbiol. 12, 252–262. doi: 10.1038/nrmicro3231 24590244

[B23] Mina M. J. McCullers J. A. Klugman K. P. (2014). Live attenuated influenza vaccine enhances colonization of *Streptococcus pneumoniae* and *Staphylococcus aureus* in mice. mBio 5, e01040-13. doi: 10.1128/mBio.01040-13 24549845 PMC3944816

[B24] Narciso A. R. Dookie R. Nannapaneni P. Normark S. Henriques-Normark B. (2025). *Streptococcus pneumoniae* epidemiology, pathogenesis and control. Nat. Rev. Microbiol. 23, 256–271. doi: 10.1038/s41579-024-01116-z 39506137

[B25] Palmu A. A. Kaijalainen T. Saukkoriipi A. Leinonen M. Kilpi T. M. (2012). Nasopharyngeal carriage of *Streptococcus pneumoniae* and pneumococcal urine antigen test in healthy elderly subjects. Scand. J. Infect. Dis. 44, 433–438. doi: 10.3109/00365548.2011.652162 22263905

[B26] Pérez-García C. Sempere J. de Miguel S. Hita S. Úbeda A. Vidal E. J. . (2024). Surveillance of invasive pneumococcal disease in Spain exploring the impact of the COVID-19 pandemic (2019-2023). J. Infection 89 (2), 106204. doi: 10.1016/j.jinf.2024.106204 38906265

[B27] Rybak A. Assad Z. Levy C. Bonarcorsi S. Béchet S. Werner A. . (2024). Age-specific resurgence in invasive pneumococcal disease incidence in the COVID-19 pandemic era and its association with respiratory virus and pneumococcal carriage dynamics: A time-series analysis. Clin. Infect. Dis. 78, 855–859. doi: 10.1093/cid/ciad746 38059538

[B28] Safadi D. E. Hitchins L. Howard A. Aley P. Bowman J. Bertran M. . (2025). Pneumococcal carriage and disease in adults in England, 2011-2019: The importance of adults as a reservoir for pneumococcus in communities. J. Infect. Dis. 231, e17–e27. doi: 10.1093/INFDIS/JIAE351 39013016 PMC11793058

[B29] Sá-Leao R. Pinto F. Aguiar S. Nunes S. Carriço J. A. Frazao N. . (2011). Analysis of invasiveness of pneumococcal serotypes and clones circulating in Portugal before widespread use of conjugate vaccines reveals heterogeneous behavior of clones expressing the same serotype. J. Clin. Microbiol. 49, 1369–1375. doi: 10.1128/JCM.01763-10 21270219 PMC3122870

[B30] Shaw D. Abad R. Amin-Chowdhury Z. Bautista A. Bennett D. Broughton K. . (2023). Trends in invasive bacterial diseases during the first 2 years of the COVID-19 pandemic: Analyses of prospective surveillance data from 30 countries and territories in the IRIS Consortium. Lancet Digit Health 5, e582–e593. doi: 10.1016/S2589-7500(23)00108-5 37516557 PMC10914672

[B31] Siegel S. J. Roche A. M. Weiser J. N. (2014). Influenza promotes pneumococcal growth during coinfection by providing host sialylated substrates as a nutrient source. Cell Host Microbe 16, 55–67. doi: 10.1016/j.chom.2014.06.005 25011108 PMC4096718

[B32] Tavares D. A. Handem S. Carvalho R. J. Paulo A. C. de Lencastre H. Hinds J. . (2019). Identification of *Streptococcus pneumoniae* by a real-time PCR assay targeting SP2020. Sci. Rep. 9 (1), 3285. doi: 10.1038/s41598-019-39791-1 30824850 PMC6397248

[B33] Therneau T. M. Grambsch P. M. (2000). Modeling Survival Data: Extending the Cox Model. New York, NY, USA: Springer. doi: 10.1007/978-1-4757-3294-8

[B34] Toor J. Echeverria-Londono S. Li X. Abbas K. Carter E. D. Clapham H. E. . (2021). Lives saved with vaccination for 10 pathogens across 112 countries in a pre-COVID-19 world. Elife 10, e67635. doi: 10.7554/eLife.67635 34253291 PMC8277373

[B35] Trzciński K. Bogaert D. Wyllie A. Chu M. L. J. N. van der Ende A. Bruin J. P. . (2013). Superiority of trans-oral over trans-nasal sampling in detecting *Streptococcus pneumoniae* colonization in adults. PloS One 8 (3), e60520. doi: 10.1371/journal.pone.0060520 23555985 PMC3610877

[B36] Weiser J. N. Ferreira D. M. Paton J. C. (2018). *Streptococcus pneumoniae*: transmission, colonization and invasion. Nat. Rev. Microbiol. 16, 355–367. doi: 10.1038/s41579-018-0001-8 29599457 PMC5949087

[B37] Wolter N. Tempia S. Cohen C. Madhi S. A. Venter M. Moyes J. . (2014). High nasopharyngeal pneumococcal density, increased by viral coinfection, is associated with invasive pneumococcal pneumonia. J. Infect. Dis. 210, 1649–1657. doi: 10.1093/infdis/jiu326 24907383

[B38] Wong A. Warren J. L. Fitch L. Perniciaro S. Dagan R. Weinberger D. M. (2025). Estimating the serotype-specific association between the concentration of vaccine-induced serum antibodies and protection against pneumococcal colonization. J. Infect. Dis. 232, e27–e34. doi: 10.1093/infdis/jiaf106 40036886 PMC12308679

[B39] Wyllie A. L. Mbodj S. Thammavongsa D. A. Hislop M. S. Yolda-Carr D. Waghela P. . (2023). Persistence of pneumococcal carriage among older adults in the community despite COVID-19 mitigation measures. Microbiol. Spectr 11 (3), e04879-22. doi: 10.1128/spectrum.04879-22 37036377 PMC10269788

[B40] Wyllie A. L. Yolda-Carr D. Hislop M. S. Mbodj S. Wurst L. Waghela P. . (2024). Contact with young children is a major risk factor for pneumococcal colonization in older adults. FEMS Microbes 5, xtae032. doi: 10.1093/femsmc/xtae032 39502507 PMC11536760

[B41] Xue L. Jing S. Zhang K. Milne R. Wang H. (2022). Infectivity versus fatality of SARS-CoV-2 mutations and influenza. Int. J. Infect. Dis. 121, 195–202. doi: 10.1016/j.ijid.2022.05.031 35584743 PMC9107628

[B42] Zhou H. Haber M. Ray S. Farley M. M. Panozzo C. A. Klugman K. P. (2012). Invasive pneumococcal pneumonia and respiratory virus co-infections. Emerg. Infect. Dis. 18, 294–297. doi: 10.3201/eid1802.102025 22305270 PMC3310442

