# Supplementary material for: Hidden burden of pneumococcal colonization in adults with viral respiratory infections: the role of influenza and RSV
Source: Front Cell Infect Microbiol. 2026 Sep 21;16:1929747. doi: 10.3389/fcimb.2026.1929747 (PMC13634830; doi:10.3389/fcimb.2026.1929747)
Supplement: Supplementary file 1 [file DataSheet1.pdf]

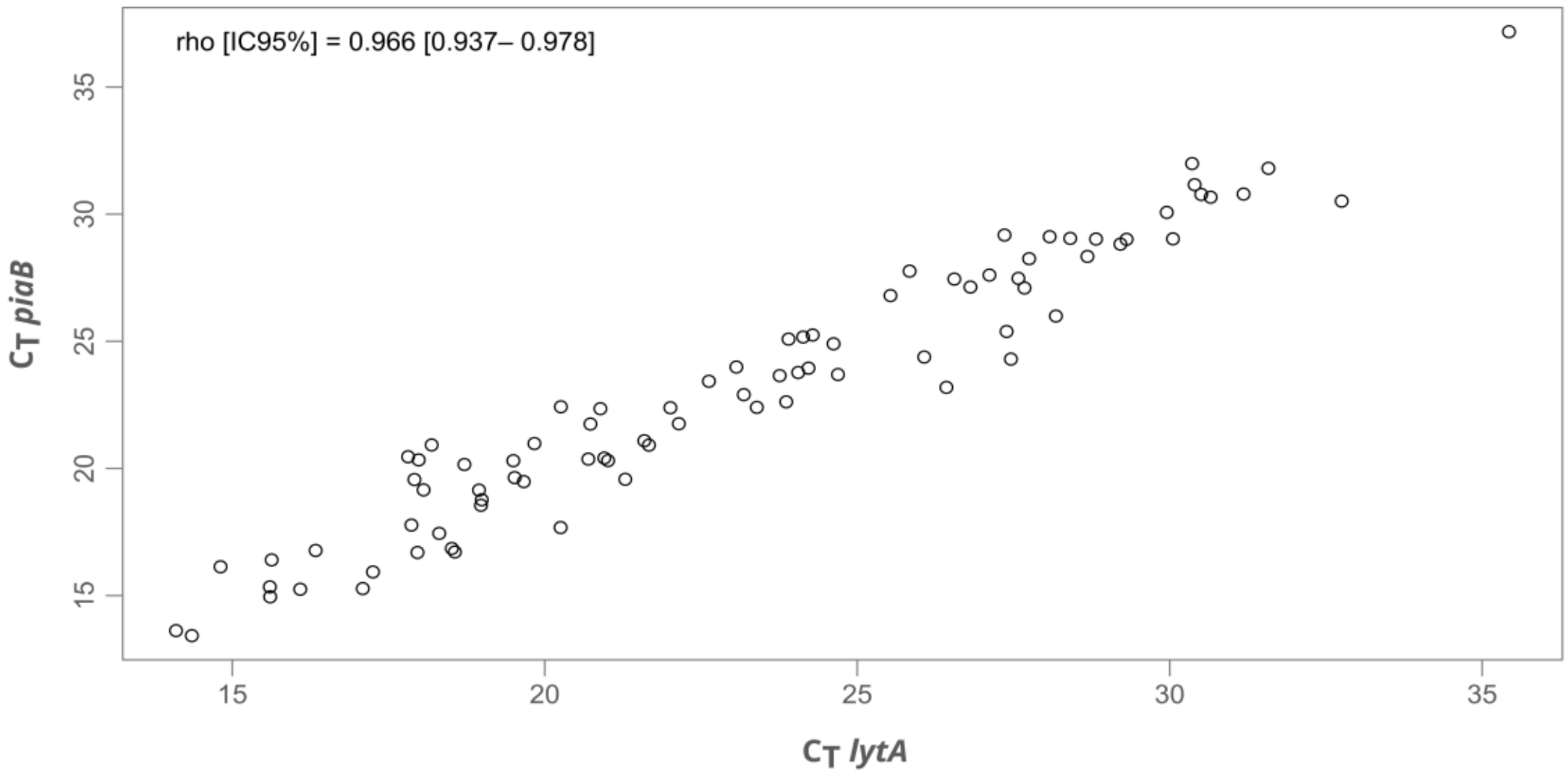

Supplementary Material, Figure 1. Correlation between *lytA* and *piaB* C_T values. Data was analysed through Spearman correlation test and CI95% via bootstrap method was calculated.

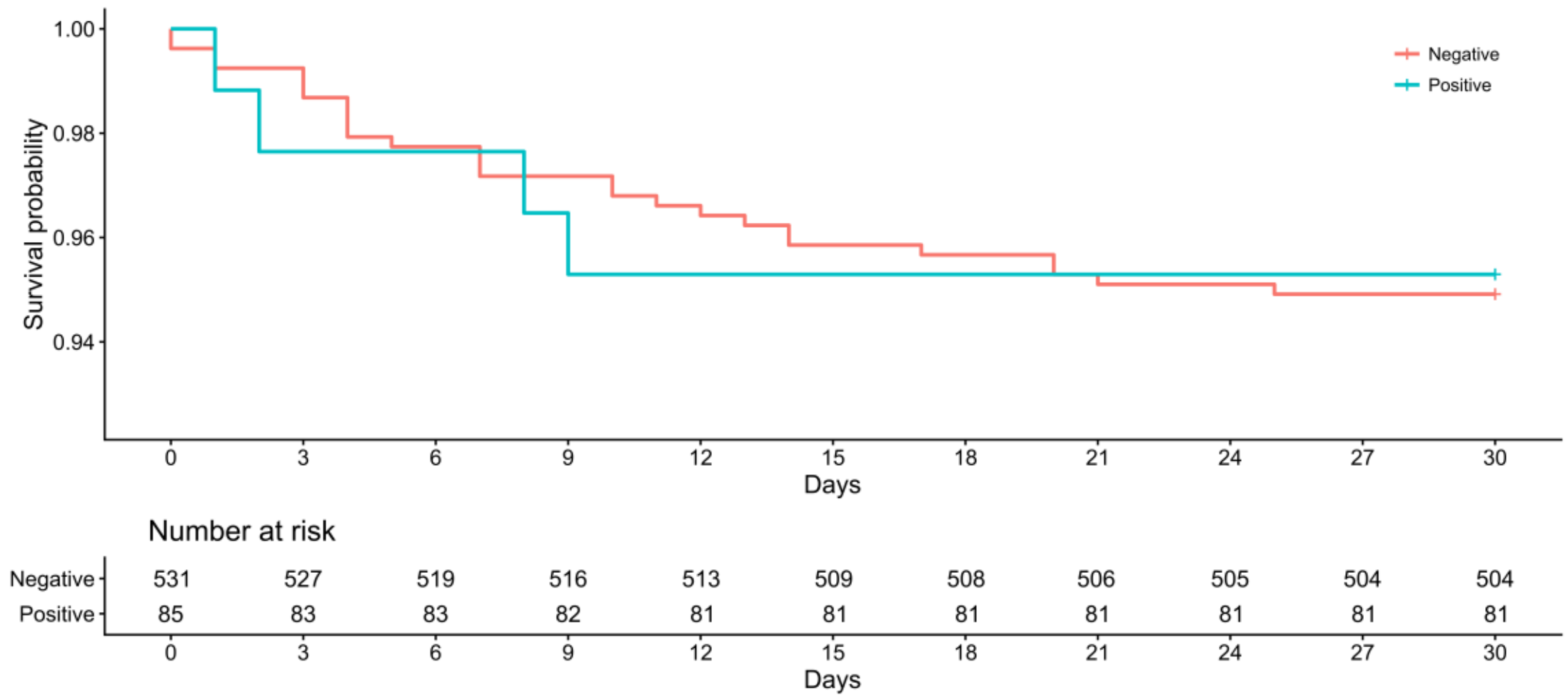

Supplementary Material, Figure 2. Kaplan–Meier survival curve by pneumococcal colonization. The lower table displays the number of patients at risk at different time points, stratified by pneumococcal colonization ("positive").
