# Supplementary material for: Hidden burden of pneumococcal colonization in adults with viral respiratory infections: the role of influenza and RSV
Source: Front Cell Infect Microbiol. 2026 Sep 21;16:1929747. doi: 10.3389/fcimb.2026.1929747 (PMC13634830; doi:10.3389/fcimb.2026.1929747)
Supplement: Supplementary file 2 [file DataSheet2.pdf]

Supplementary Material Table S1. Real-time PCR primers and probes for the detection of *Streptococcus pneumoniae* .

Gene		Primer	Concentration	Reference
lytA	Foward	5'-ACGCAATCTAGCAGATGAAGCA-3'	150 nM	Carvalho Mda G, Tondella ML, McCaustland K, *et al* . Evaluation and improvement of real-time PCR assays targeting *lytA, ply* , and *psaA* genes for detection of pneumococcal DNA. *J Clin Microbiol* . 2007;45(8):2460-2466. doi:10.1128/JCM.02498-06
	Reverse	5'-TCGTGCGTTTTAATTCCAGCT-3'	150 nM	
	Probe	5'-FAM-GCCGAAAACGCTTGATACAGGGAG-3'-BHQ1	75 nM	
PiaB	Foward	5'-CATTGGTGGCTTAGTAAGTGCAA-3'	150 nM	Trzciński K, Bogaert D, Wyllie A, *et al* . Superiority of trans-oral over trans-nasal sampling in detecting *Streptococcus pneumoniae* colonization in adults. *PLoS One* . 2013;8(3):e60520. doi:10.1371/journal.pone.0060520
	Reverse	5'-TACTAACACAAGTTCCTGATAAGGCAAGT-3'	150 nM	
	Probe	5'-FAM-TGTAAGCGGAAAAGCAGGCCTTACCC-3'-BHQ1	175 nM	
SP2020	Foward	5’-TAAACAGTTTGCCTGTAGTCG-3’	150 nM	Tavares DA, Handem S, Carvalho RJ, *et al* . Identification of *Streptococcus pneumoniae* by a real-time PCR assay targeting *SP2020* . *Sci Rep* . 2019;9(1):3285. Published 2019 Mar 1. doi:10.1038/s41598-019-39791-1
	Reverse	5’-CCCGGATATCTCTTTCTGGA-3’	150 nM	
	Probe	5’-FAM-AACCTTTGTTCTCTCTCGTGGCAGCTCAA-BHQ-3’	75 nM	

Supplementary Material, Table S2. Clinical data by type of viral infection and age group.

	**All episodes**						**<65 years old**						**≥65 years old**					
	Influenza A n=391	SARS-CoV-2 n=162	RSV n=28	>1 virus n=35	All n=616	*p* -value	Influenza A n=159	SARS-CoV-2 n=46	RSV n=12	>1 virus n=12	All n=229	*p* -value	Influenza A n=232	SARS-CoV-2 n=116	RSV n=16	>1 virus n=23	All n=387	*p* -value
Age, Median [Q1;Q3]	70.0 [51.0;80.0]	75.5 [61.5;83.0]	69.0 [56.5;74.0]	75.0 [57.0;85.0]	72.0 [55.0;81.0]	**0.002**	49.0 [40.0;56.0]	54.0 [48.0;59.8]	54.5 [32.2;59.2]	51.0 [42.2;56.5]	50.0 [42.0;58.0]	**0.049**	79.0 [72.0;85.0]	80.0 [74.8;86.2]	74.0 [71.5;77.0]	80.0 [76.0;87.5]	79.0 [73.0;85.0]	**0.029**
Female Sex	191 (48.8%)	79 (48.8%)	13 (46.4%)	17 (48.6%)	300 (48.7%)	0.996	75 (47.2%)	18 (39.1%)	4 (33.3%)	7 (58.3%)	104 (45.4%)	0.485	116 (50.0%)	61 (52.6%)	9 (56.2%)	10 (43.5%)	196 (50.6%)	0.829
Current smoking	163 (41.7%)	66 (40.7%)	13 (46.4%)	12 (34.3%)	254 (41.2%)	0.787	65 (40.9%)	23 (50.0%)	7 (58.3%)	5 (41.7%)	100 (43.7%)	0.509	98 (42.2%)	43 (37.1%)	6 (37.5%)	7 (30.4%)	154 (39.8%)	0.612
Alcohol abuse	53 (13.6%)	11 (6.8%)	2 (7.1%)	3 (8.6%)	69 (11.2%)	0.120	21 (13.2%)	5 (10.9%)	0 (0.0%)	1 (8.3%)	27 (11.8%)	0.754	32 (13.8%)	6 (5.2%)	2 (12.5%)	2 (8.7%)	42 (10.9%)	0.074
Number of comorbidities						0.297						0.252						0.782
0	105 (26.9%)	34 (21.0%)	5 (17.9%)	3 (8.6%)	147 (23.9%)	-	76 (47.8%)	16 (34.8%)	4 (33.3%)	2 (16.7%)	98 (42.8%)	-	29 (12.5%)	18 (15.5%)	1 (6.2%)	1 (4.3%)	49 (12.7%)	-
1	96 (24.6%)	43 (26.5%)	9 (32.1%)	12 (34.3%)	160 (26.0%)	-	33 (20.8%)	14 (30.4%)	5 (41.7%)	5 (41.7%)	57 (24.9%)	-	63 (27.2%)	29 (25.0%)	4 (25.0%)	7 (30.4%)	103 (26.6%)	-
2	101 (25.8%)	52 (32.1%)	6 (21.4%)	11 (31.4%)	170 (27.6%)	-	32 (20.1%)	11 (23.9%)	1 (8.3%)	2 (16.7%)	46 (20.1%)	-	69 (29.7%)	41 (35.3%)	5 (31.2%)	9 (39.1%)	124 (32.0%)	-
>2	89 (22.8%)	33 (20.4%)	8 (28.6%)	9 (25.7%)	139 (22.6%)	-	18 (11.3%)	5 (10.9%)	2 (16.7%)	3 (25.0%)	28 (12.2%)	-	71 (30.6%)	28 (24.1%)	6 (37.5%)	6 (26.1%)	111 (28.7%)	-
Diabetes	98 (25.1%)	38 (23.5%)	6 (21.4%)	10 (28.6%)	152 (24.7%)	0.895	15 (9.4%)	3 (6.5%)	2 (16.7%)	1 (8.3%)	21 (9.2%)	0.668	83 (35.8%)	35 (30.2%)	4 (25.0%)	9 (39.1%)	131 (33.9%)	0.587
Chronic liver disease	17 (4.3%)	12 (7.4%)	1 (3.6%)	4 (11.4%)	34 (5.5%)	0.160	6 (3.8%)	6 (13.0%)	0 (0.0%)	3 (25.0%)	15 (6.6%)	**0.009**	11 (4.7%)	6 (5.2%)	1 (6.2%)	1 (4.3%)	19 (4.9%)	0.935
Chronic heart disease	136 (34.8%)	35 (21.6%)	9 (32.1%)	13 (37.1%)	193 (31.3%)	**0.020**	22 (13.8%)	4 (8.7%)	1 (8.3%)	1 (8.3%)	28 (12.2%)	0.855	114 (49.1%)	31 (26.7%)	8 (50.0%)	12 (52.2%)	165 (42.6%)	**0.001**
COPD	74 (18.9%)	24 (14.8%)	2 (7.1%)	9 (25.7%)	109 (17.7%)	0.171	11 (6.9%)	2 (4.3%)	1 (8.3%)	2 (16.7%)	16 (7.0%)	0.378	63 (27.2%)	22 (19.0%)	1 (6.2%)	7 (30.4%)	93 (24.0%)	0.102
Bronchiectasis	8 (2.0%)	3 (1.9%)	1 (3.6%)	0 (0.0%)	12 (1.9%)	0.723	2 (1.3%)	0 (0.0%)	0 (0.0%)	0 (0.0%)	2 (0.9%)	1.0	6 (2.6%)	3 (2.6%)	1 (6.2%)	0 (0.0%)	10 (2.6%)	0.552
Asthma	53 (13.6%)	12 (7.4%)	4 (14.3%)	7 (20.0%)	76 (12.3%)	0.077	22 (13.8%)	4 (8.7%)	2 (16.7%)	4 (33.3%)	32 (14.0%)	0.159	31 (13.4%)	8 (6.9%)	2 (12.5%)	3 (13.0%)	44 (11.4%)	0.283
Chronic renal failure	75 (19.2%)	23 (14.2%)	3 (10.7%)	11 (31.4%)	112 (18.2%)	0.067	16 (10.1%)	4 (8.7%)	0 (0.0%)	3 (25.0%)	23 (10.0%)	0.242	59 (25.4%)	19 (16.4%)	3 (18.8%)	8 (34.8%)	89 (23.0%)	0.129
Malignancies	54 (13.8%)	43 (26.5%)	8 (28.6%)	6 (17.1%)	111 (18.0%)	**0.002**	22 (13.8%)	11 (23.9%)	3 (25.0%)	4 (33.3%)	40 (17.5%)	0.095	32 (13.8%)	32 (27.6%)	5 (31.2%)	2 (8.7%)	71 (18.3%)	**0.004**
Immunosupression	82 (21.0%)	70 (43.2%)	15 (53.6%)	8 (22.9%)	175 (28.4%)	**<0.001**	39 (24.5%)	18 (39.1%)	5 (41.7%)	5 (41.7%)	67 (29.3%)	0.111	43 (18.5%)	52 (44.8%)	10 (62.5%)	3 (13.0%)	108 (27.9%)	**<0.001**
HIV	6 (1.5%)	1 (0.6%)	0 (0.0%)	1 (2.9%)	8 (1.3%)	0.557	6 (3.8%)	1 (2.2%)	0 (0.0%)	1 (8.3%)	8 (3.5%)	0.676	0 (0.0%)	0 (0.0%)	0 (0.0%)	0 (0.0%)	0 (0.0%)	-
Clinical presentation																		
Mild disease	31 (7.9%)	23 (14.2%)	1 (3.6%)	0 (0.0%)	55 (8.9%)	**0.015**	12 (7.5%)	9 (19.6%)	1 (8.3%)	0 (0.0%)	22 (9.6%)	0.086	19 (8.2%)	14 (12.1%)	0 (0.0%)	0 (0.0%)	33 (8.5%)	0.173
Pneumonia	76 (19.4%)	34 (21.0%)	6 (21.4%)	10 (28.6%)	126 (20.5%)	0.635	31 (19.5%)	9 (19.6%)	4 (33.3%)	4 (33.3%)	48 (21.0%)	0.411	45 (19.4%)	25 (21.6%)	2 (12.5%)	6 (26.1%)	78 (20.2%)	0.735
Hospitalization	157 (40.2%)	50 (30.9%)	13 (46.4%)	13 (37.1%)	233 (37.8%)	0.163	46 (28.9%)	10 (21.7%)	4 (33.3%)	4 (33.3%)	64 (27.9%)	0.681	111 (47.8%)	40 (34.5%)	9 (56.2%)	9 (39.1%)	169 (43.7%)	0.077
ICU admission	8 (2.0%)	0 (0.0%)	1 (3.6%)	0 (0.0%)	9 (1.5%)	0.146	6 (3.8%)	0 (0.0%)	0 (0.0%)	0 (0.0%)	6 (2.6%)	0.664	2 (0.9%)	0 (0.0%)	1 (6.2%)	0 (0.0%)	3 (0.8%)	0.126
Respiratory failure	71 (18.2%)	36 (22.2%)	9 (32.1%)	6 (17.1%)	122 (19.8%)	0.251	17 (10.7%)	5 (10.9%)	3 (25.0%)	2 (16.7%)	27 (11.8%)	0.379	54 (23.3%)	31 (26.7%)	6 (37.5%)	4 (17.4%)	95 (24.5%)	0.455
Pleural effusion	6 (1.5%)	3 (1.9%)	1 (3.6%)	1 (2.9%)	11 (1.8%)	0.423	2 (1.3%)	1 (2.2%)	0 (0.0%)	0 (0.0%)	3 (1.3%)	0.667	4 (1.7%)	2 (1.7%)	1 (6.2%)	1 (4.3%)	8 (2.1%)	0.271
Sepsis	5 (1.3%)	2 (1.2%)	1 (3.6%)	2 (5.7%)	10 (1.6%)	0.135	3 (1.9%)	0 (0.0%)	0 (0.0%)	0 (0.0%)	3 (1.3%)	1.0	2 (0.9%)	2 (1.7%)	1 (6.2%)	2 (8.7%)	7 (1.8%)	**0.035**
Vaccination status																		
SARS-CoV-2	351 (89.8%)	147 (90.7%)	27 (96.4%)	32 (91.4%)	557 (90.4%)	0.811	130 (81.8%)	39 (84.8%)	11 (91.7%)	10 (83.3%)	190 (83.0%)	0.876	221 (95.3%)	108 (93.1%)	16 (100.0%)	22 (95.7%)	367 (94.8%)	0.764
Seasonal Influenza	151 (38.6%)	75 (46.3%)	10 (35.7%)	9 (25.7%)	245 (39.8%)	0.103	19 (11.9%)	13 (28.3%)	2 (16.7%)	3 (25.0%)	37 (16.2%)	0.159	132 (56.9%)	62 (53.4%)	8 (50.0%)	6 (26.1%)	208 (53.7%)	0.137
PCV	59 (15.1%)	40 (24.7%)	3 (10.7%)	6 (17.1%)	108 (17.5%)	**0.048**	19 (11.9%)	9 (19.6%)	0 (0.0%)	3 (25.0%)	31 (13.5%)	0.150	40 (17.2%)	31 (26.7%)	3 (18.8%)	3 (13.0%)	77 (19.9%)	0.182
30-day mortality	15 (3.8%)	11 (6.8%)	1 (3.6%)	4 (11.4%)	31 (5.0%)	0.124	2 (1.3%)	2 (4.3%)	0 (0.0%)	0 (0.0%)	4 (1.7%)	0.499	13 (5.6%)	9 (7.8%)	1 (6.2%)	4 (17.4%)	27 (7.0%)	0.171

Data is shown as number of cases and percentage, otherwise stated. Statistically significant values are highlighted in bold. Immunosuppression was defined as the presence of one or more of the following conditions: active HIV infection and/or AIDS, receipt of antineoplastic chemotherapy within the previous 3 months, use of immunosuppressive drugs in the previous 6 months, or primary immunodeficiency disorders. >1 virus refers to the detection of two or more viruses in a single sample. COPD: chronic obstructive pulmonary disease. PCV: pneumococcal conjugate vaccine.

Supplementary Material, Table S3. Logistic regression models for *Streptococcus pneumoniae* colonization.

All individuals

Variables	Model 1			Model 2			Model 3		
	Odds Ratio	95% CI	*p*	Odds Ratio	95% CI	*p*	Odds Ratio	95% CI	*p*
Age (years)	0.99	0.98 – 1.01	0.288	0.99	0.98 – 1.01	0.273	0.99	0.98 – 1.00	0.197
Sex (male)	0.73	0.44 – 1.19	0.203	0.73	0.45 – 1.20	0.213	0.74	0.45 – 1.21	0.228
Current smoking	1.50	0.87 – 2.56	0.139	1.64	1.00 – 2.70	0.051	1.49	0.87 – 2.53	0.143
COPD	1.53	0.80 – 2.88	0.193	-	-	-	1.54	0.81 – 2.89	0.181
Comorbidities (no COPD)[>2 vs. 0-2]	1.09	0.57 – 1.98	0.796	-	-	-	1.23	0.66 – 2.19	0.492
Comorbidities[>2 vs. 0-2]	-	-	-	1.18	0.66 – 2.05	0.575	-	-	-
Viral infection									
**Influenza A**	**3.52**	**1.30 – 9.17**	**0.011**	**3.34**	**1.21 – 8.73**	**0.015**	**3.33**	**1.25 – 8.51**	**0.013**
SARS-CoV-2	2.01	0.82 – 4.54	0.105	1.89	0.75 – 4.30	0.147	2.03	0.85 – 4.50	0.094
**RSV**	**4.04**	**1.11 – 13.50**	**0.027**	**3.69**	**1.01 – 12.29**	**0.038**	**3.62**	**1.01 – 11.85**	**0.039**
Vaccines									
Seasonal Influenza	1.08	0.61 – 1.88	0.798	-	-	-	-	-	-
SARS-CoV-2	0.60	0.29 – 1.33	0.187	-	-	-	-	-	-
PCV	1.72	0.92 – 3.13	0.082	1.63	0.89 – 2.90	0.103	-	-	-

Individuals <65 years old

Variables	Model 1			Model 2			Model 3		
	Odds Ratio	95% CI	*p*	Odds Ratio	95% CI	*p*	Odds Ratio	95% CI	*p*
Age (years)	1.00	0.97 – 1.04	0.977	0.99	0.96 – 1.03	0.699	0.99	0.96 – 1.03	0.712
**Sex (male)**	**0.43**	**0.19 – 0.90**	**0.028**	**0.43**	**0.20 – 0.89**	**0.026**	**0.47**	**0.22 – 0.97**	**0.044**
Smoking	1.10	0.48 – 2.42	0.824	1.04	0.48 – 2.21	0.922	1.02	0.46 – 2.22	0.955
COPD	1.67	0.35 – 6.85	0.489	-	-	-	1.68	0.38 – 6.40	0.460
Comorbidities (no COPD)[>2 vs. 0-2]	1.41	0.37 – 4.75	0.591	-	-	-	1.76	0.56 – 4.94	0.303
Comorbidities[>2 vs. 0-2]	-	-	-	1.62	0.49 – 4.83	0.400	-	-	-
Viral infection									
Influenza A	3.70	0.69 – 18.90	0.114	2.94	0.51 – 15.20	0.200	3.73	0.75 – 18.15	0.098
SARS-CoV-2	2.00	0.45 – 7.64	0.328	1.47	0.29 – 5.74	0.598	1.99	0.48 – 7.15	0.305
RSV	2.38	0.25 – 17.47	0.406	2.05	0.21 – 14.72	0.494	2.31	0.25 – 15.91	0.412
Vaccines									
Seasonal Influenza	0.72	0.20 – 2.18	0.579	-	-	-	-	-	-
**SARS-CoV-2**	**0.36**	**0.15 – 0.88**	**0.023**	-	-	-	-	-	-
PCV	1.94	0.54 – 6.33	0.286	1.51	0.46 – 4.44	0.468	-	-	-

Individuals >=65 years old

Variables	Model 1			Model 2			Model 3		
	Odds Ratio	95% CI	*p*	Odds Ratio	95% CI	*p*	Odds Ratio	95% CI	*p*
Age (years)	1.03	0.98 – 1.07	0.253	1.03	0.99 – 1.08	0.161	1.03	0.99 – 1.08	0.169
Sex (male)	0.95	0.45 – 1.96	0.883	0.95	0.46 – 1.96	0.886	0.95	0.46 – 1.96	0.883
**Smoking**	2.06	0.92 – 4.73	0.082	**2.41**	**1.15 – 5.20**	**0.022**	2.12	0.95 – 4.83	0.070
COPD	1.44	0.67 – 3.03	0.337	-	-	-	1.40	0.66 – 2.92	0.370
Comorbidities (no COPD)[>2 vs. 0-2]	1.01	0.45 – 2.09	0.986	-	-	-	1.10	0.50 – 2.25	0.805
Comorbidities[>2 vs. 0-2]	-	-	-	1.10	0.55 – 2.13	0.782	-	-	-
Viral infection									
Influenza A	2.94	0.76 – 10.38	0.101	2.99	0.79 – 10.23	0.088	2.61	0.69 – 8.84	0.133
SARS-CoV-2	1.87	0.53 – 5.64	0.291	1.86	0.55 – 5.41	0.277	1.76	0.52 – 5.05	0.323
**RSV**	**5.56**	**1.03 – 27.31**	**0.037**	**5.43**	**1.03 – 25.76**	**0.036**	**4.93**	**0.94 – 23.06**	**0.047**
Vaccines									
Seasonal Influenza	1.18	0.59 – 2.37	0.642	-	-	-	-	-	-
SARS-CoV-2*	-	-	-	-	-	-	-	-	-
PCV	1.76	0.81 – 3.64	0.137	1.78	0.84 – 3.61	0.119	-	-	-

*not included due to insufficient values

Age was included in the model as a continuous variable. Comorbidities (no COPD)[>2 vs. 0-2] refers to the number of comorbidities excluding COPD, which was included in the model as individual variable. The variable Comorbidities[>2 vs. 0-2] includes COPD.
COPD: chronic obstructive pulmonary disease. PCV: pneumococcal conjugate vaccine.

Supplementary Material, Table S4. Cox regression models for 30-day mortality.

Variables	Model 1			Model 2			Model 3		
	Hazard ratio	95% CI	*p*	Hazard ratio	95% CI	*p*	Hazard ratio	95% CI	*p*
Age (years)	1.01	0.98– 1.04	0.6	1.02	0.98– 1.05	0.4	1.02	0.99– 1.05	0.2
Gender (male)	1.47	0.70– 3.10	0.3	1.48	0.71– 3.09	0.3	1.49	0.71– 3.11	0.3
Comorbidities									
**Chronic Heart Disease**	**3.06**	**1.29– 7.27**	**0.011**	**2.81**	**1.22– 6.47**	**0.015**	**2.82**	**1.22– 6.51**	**0.015**
Malignancies	1.15	0.45– 2.96	0.8	1.16	0.46– 2.93	0.8	1.30	0.51– 3.30	0.6
**Immunosuppression**	**2.77**	**1.22– 6.29**	**0.015**	**2.70**	**1.18– 6.16**	**0.018**	**2.27**	**1.01– 5.08**	**0.046**
Clinical presentation									
**Respiratory failure**	**4.83**	**1.93– 12.1**	**<0.001**	-	-	-	-	-	-
Mild disease	1.92	0.42– 8.85	0.4	-	-	-	-	-	-
Viral Infection									
Influenza A	3.63	1.00– 13.2	0.051	1.95	0.54– 7.02	0.3	1.70	0.49– 5.95	0.4
**SARS-CoV-2**	**6.08**	**1.86– 19.8**	**0.003**	**4.38**	**1.36– 14.1**	**0.013**	**3.49**	**1.12– 10.9**	**0.031**
RSV	2.39	0.41– 13.9	0.3	1.86	0.31– 11.2	0.5	1.74	0.29– 10.3	0.5
Hospitalization									
≤5 days	1.52	0.41– 5.63	0.5	3.38	1.04– 11.0	0.044	3.14	0.97– 10.2	0.057
**>5 days**	**10.9**	**1.31– 90.2**	**0.027**	**24.1**	**3.15– 184**	**0.002**	**22.0**	**2.88– 168**	**0.003**
Vaccines									
SARS-CoV-2	2.99	0.38– 23.6	0.3	2.70	0.35– 20.9	0.3	-	-	-
Influenza A	1.49	0.68– 3.26	0.3	1.31	0.60– 2.88	0.5	-	-	-
PCV	0.54	0.21– 1.38	0.2	0.51	0.19– 1.31	0.2	-	-	-
Streptococcus pneumoniae colonization	0.68	0.22– 2.05	0.5	0.83	0.28– 2.53	0.7	0.81	0.27– 2.43	0.7

To meet the proportional hazards assumption, length of hospitalization was stratified into two periods: 0 to 5 days and >5 days. Statistically significant values are highlighted in bold. Immunosuppression was defined as the presence of one or more of the following conditions: active HIV infection and/or AIDS, receipt of antineoplastic chemotherapy within the previous 3 months, use of immunosuppressive drugs in the previous 6 months, or primary immunodeficiency disorders. PCV: pneumococcal conjugate vaccine.
